# CirGO: an alternative circular way of visualising gene ontology terms

**DOI:** 10.1186/s12859-019-2671-2

**Published:** 2019-02-18

**Authors:** Irina Kuznetsova, Artur Lugmayr, Stefan J. Siira, Oliver Rackham, Aleksandra Filipovska

**Affiliations:** 1 Harry Perkins Institute of Medical Research, Nedlands, Western Australia 6009 Australia; 2 Visualisation and Interactive Media (VisLab), Curtin University, Perth, 6102 Australia; 3 School of Molecular Sciences, University of Western Australia, Nedlands, 6009 Australia

**Keywords:** Gene ontology terms, Visualisation, Data organisation, Python, Bioinformatics

## Abstract

**Background:**

Prioritisation of gene ontology terms from differential gene expression analyses in a two-dimensional format remains a challenge with exponentially growing data volumes. Typically, gene ontology terms are represented as tree-maps that enclose all data into defined space. However, large datasets make this type of visualisation appear cluttered and busy, and often not informative as some labels are omitted due space limits, especially when published in two-dimensional (2D) figures.

**Results:**

Here we present an open source CirGO (**Cir**cular **G**ene **O**ntology) software that visualises non-redundant two-level hierarchically structured ontology terms from gene expression data in a 2D space. Gene ontology terms based on statistical significance were summarised with a semantic similarity algorithm and grouped by hierarchical clustering. This software visualises the most enriched gene ontology terms in an informative, comprehensive and intuitive format that is achieved by organising data from the most relevant to the least, as well as the appropriate use of colours and supporting information. Additionally, CirGO is an easy to use software that supports researchers with little computational background to present their gene ontology data in a publication ready format.

**Conclusions:**

Our easy to use open source CirGO Python software package provides biologists with a succinct presentation of terms and functions that are most represented in a specific gene expression data set in a visually appealing 2D format (e.g. for reporting research results in scientific articles). CirGO is freely available at https://github.com/IrinaVKuznetsova/CirGO.git.

**Electronic supplementary material:**

The online version of this article (10.1186/s12859-019-2671-2) contains supplementary material, which is available to authorized users.

## Background

Advances in next generation sequencing technologies have led to the generation of large volumes of digital data. Consequently, this has resulted in the development of bioinformatics tools that use knowledge from various computational and biological disciplines to display these large amounts of data in accessible ways. RNA sequencing (RNA-Seq) has become the gold standard for analysing gene expression changes in diverse biological organisms and systems [1–5]. RNA-Seq datasets are analysed and processed according to algorithms and statistical techniques that identify significantly altered transcripts from whole transcriptomes [3]. The identified changes in transcripts need to be organised and prioritised in categories according to their functional properties and relationships using Gene Ontology (GO) enrichment analyses.

The GO project initiative aims to organise current biological knowledge of genes and gene products in a structured and consistent way. The main purpose of the GO project is to: (1) provide a uniform vocabulary; (2) control and provide relationships of genes and gene products; and (3) provide accessible data structures that can be updated, accessed, or retrieved at any time due to the dynamic changing nature of the biological field [6–9]. The GO project comprises of two main concepts: the GO ontology and GO annotations [6–8, 10, 11]. The GO ontology entries are called GO terms that describe gene functions and show relationships between them. GO terms have a hierarchical directed acyclic graph structure that is similar to the structure of hierarchical trees [6–10]. A descendant (child) in the hierarchical directed acyclic graph can have multiple ancestors (parents), whereas in the case of the hierarchal layout the descendant can have only one ancestor. Importantly, the GO developers offer three GO editions such as GO-basic, GO, and GO-plus, where only the GO-basic version has an acyclic graph structure [6, 7]. More detailed description on the GO and GO-plus editions can be found in Chapter 11 of the Gene Ontology handbook  [6] or GO website [7]. The GO annotations on the other hand, signify an association of a gene to a specific GO term. In addition, GO terms are categorised as three distinct gene functions: molecular function, cellular component, and biological process [6–9]. The GO data is regularly curated by the GO consortium and can be found at the GO website [7]*.*

GOs are commonly used to interpret results from high-throughput experiments by using a process called enrichment analysis. GO enrichment analyses generate GO terms based on statistically significant changes in gene expression or proteomic data [6, 8]. The identified GO terms that represent the statistically significant changes in datasets are then visualised. Frequently the number of GO terms in gene expression datasets are large, and it is currently a challenge to provide a non-redundant and biologically succinct list of GO terms. Therefore, visualisation methods aim to summarise and help to reveal identified patterns within the data in a comprehensive and biologically meaningful manner. A broad range of visualisation techniques have been developed to allow researchers to explore GO terms related to their data. These rely on two and three dimensional (2D/3D) graphic images, web-based presentations, or interactive visualisation to gain new insights. The traditional and intuitive way of visualising a hierarchical directed acyclic graph makes use of tree-based diagrams. GoMiner [12], RamiGO [13], Gorilla [14] and AmiGO [15] are GO enrichment tools, where GO enrichment analysis for the AmiGO software is provided by the PANTHER tool [16]. These tools utilise tree diagrams to represent GO terms, although some of these have not been updated for current use and produce images that are not practical for research publications.

To provide an example, GOrilla is web-based software used for GO enrichment analysis, that is capable of visualising partial hierarchy of GO terms as a tree diagram, where coloured boxes represent significantly enriched terms [6, 14]. However, it is often impractical to present a tree diagram in a 2D space with large datasets, particularly when it comes to publishing and presenting of the data in scientific journals and at meetings. This shortcoming also relates to other methods that utilise space-filling techniques, which omit important details such as labels when presented. For example, the method proposed by Supek and colleagues [17] aims to summarise a list of provided GO terms by removing redundant terms. Their tool called REVIGO, applies the semantic similarity method accompanied with the neighbor-joining clustering approach that results in two-level hierarchical structured data [6, 17–20]. One level represents an ancestor, and the secondary levels are related and similar in meaning descendants. In addition, REVIGO offers visualisation of results as a tree-map plot lacking important details that we discuss within this publication.

The organisation of data into two-level hierarchical modes leads to the use of space-filling visualising techniques. Space-filling techniques were developed to enable all provided data to fit into a defined space. Tree-maps belong to a space-filling technique that display hierarchical data as nested rectangles [21]. They provide a good overview of results, however large datasets make space-filling techniques less informative. Some information, such as labels, is often omitted if they cannot fit into a defined space. Additionally, the rectangular shapes in tree-maps are less intuitive when estimating their size within the dataset.

We used a two-level hierarchical format generated by REVIGO software to develop a 2D visualisation of GO terms. We considered, the most advantageous features of existing visualisation methods, to make images  more comprehensible [22]. Our CirGO method enables the visualisation of GO terms as: (1) a neat and simple image that was achieved by properly organising GO terms on a circular visualisation plot; (2) preservation of details as such as all labels with the same font size, representation of each cluster by its own colour, sorting of the resulting data from the most significant to the least significant in a clockwise way, a legend that provides supporting information for a slice size, and a colour based grouping to correlate a parent label to a group; (3) an intuitive, self-explanatory image of the most relevant findings, where the relevance is defined by statistical significance (such as *p*-values); (4) an informative, and detailed visualisation of multiple different parameters as “parent”, “child” labels, and slice proportion; (5) the use of colours and colour gradients as visual aids to guide viewers to the most represented GO terms.

## Implementation

**Fig. 1:**
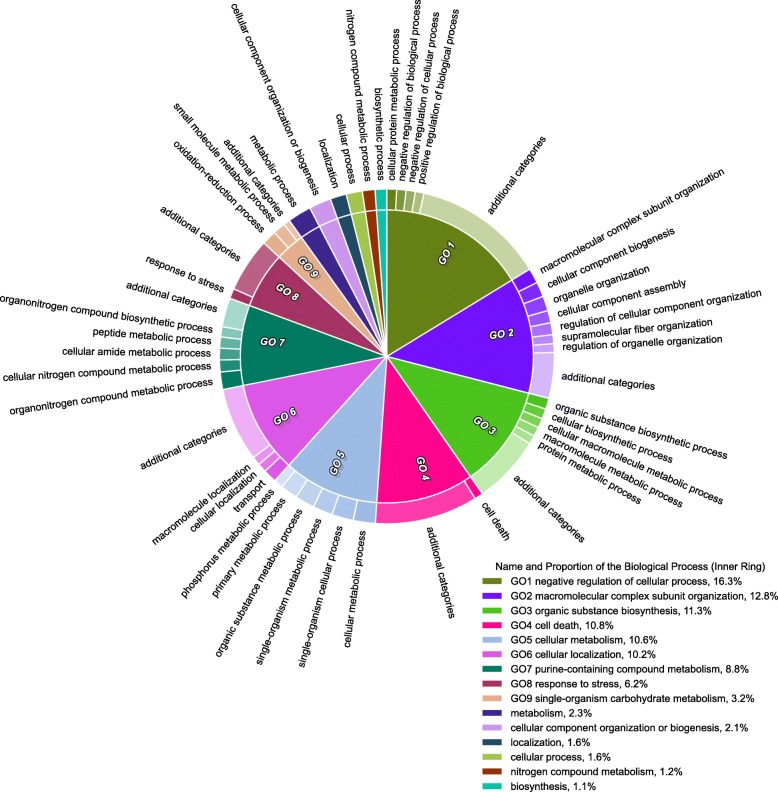
Example of CirGO image plot

CirGO is a visualisation software that provides users with a static 2D visualisation (Fig. 1) of GO terms and can be used for publishing and presentations. The visualisation script was developed as a Python package and uses Python 2.7.14, Matplotlib 2.1.0 [23] and the GUI components with TkInter (Tcl/Tk) [24], and is freely available at [25].

**Fig. 2:**
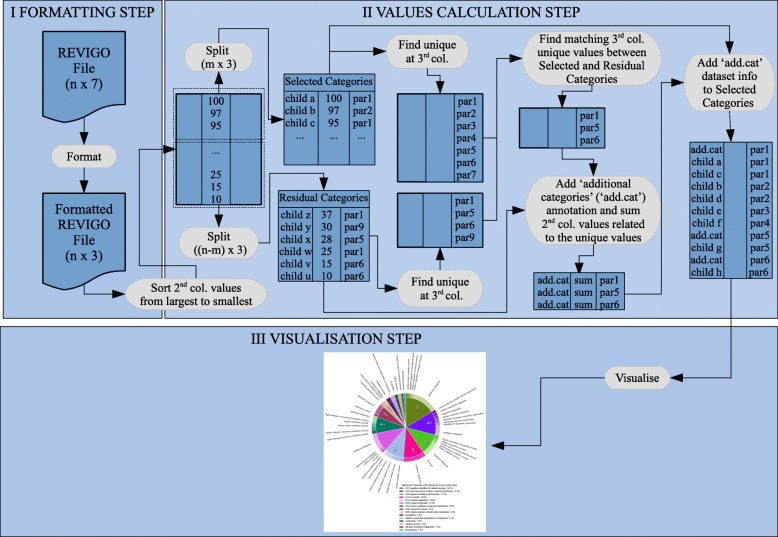
Schematic representation of the CirGO algorithm workflow. The algorithm workflow consists of three main steps: I. Formatting, II. Values calculation, and III. Visualisation. Main parameters are represented on the image as follows: n- represents number of input file rows; m- represents number of categories that will be visualised

The CirGO visualisation algorithm consists of the following three basic steps, which are also illustrated in Fig. 2.

- Formatting step: converting and preparing a REVIGO file to a processing file;
- Values calculation step: calculating, and organising values required for plotting;
- Visualisation step: visualising the data and generating the final image.

The algorithm converts a regular REVIGO file into an intermediate file, which is used to create an image as shown in Fig. 2. The final plot represents a two-layer full hierarchical structure, and consists of an inner ring, and outer ring. The inner ring represents parent records, and the outer ring child records. Precise algorithm descriptions and a tutorial on how to use CirGO software are available in the Additional file 1 and GitHub page at [25].

GO categories can be determined from a provided gene list for any organism studied that is supported by functional annotation software, such as DAVID [26]. As an example, we present a list of two-level hierarchically structured GO terms that was obtained from the REVIGO Web server, which applies semantic similarity algorithm and a neighbor-joining approach to cluster data [17, 27]. The output file was formatted with fields designated as “representative”, “description”, and the absolute value of the “log10pval”, that define parent and child records as well as the slice size, respectively.

Initially, the input data is sorted based on the absolute value of the log10pval column from the largest to smallest value. Then, a number, m, of desired categories (or number of child categories) is selected for the visualisation, and the input data is subset to this number. Unique parent records that represent the inner circle categories are extracted. All log10 *p*-values that do not fall into the m desired categories (the largest p-values from the significant range dataset) are summed up, annotated as “additional categories”, and assigned to the related inner circle categories respectively. This process results in the addition of a number of slices to a number m of desired categories. All outer ring values are sorted from the largest to smallest for each represented slice. Although combining all the small values in this category increases each slice size, it is placed within the output in the position of the smallest value, so that it corresponds to their statistical significance.

## Results and discussion

### Comparison to tree-maps

**Fig. 3:**
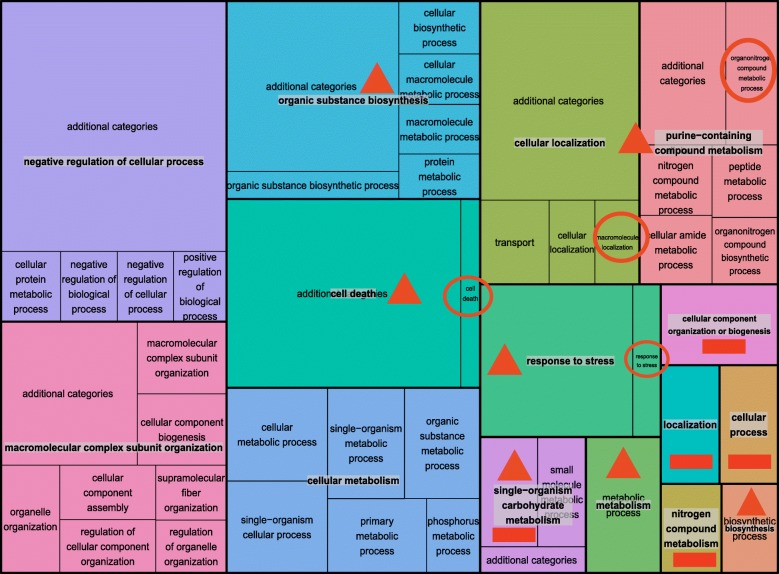
Example of a tree-map visualisation plot. The circles indicate inconsistency in the font labels. The rectangles indicate absence of child labels. The triangles indicate overlap between parent and child labels

The aim of the CirGO software was to generate visualisation that summarises GO terms from gene expression datasets in 2D space. We compared the CirGO visualisation performance (Fig. 1) to tree-maps (Fig. 3), where the input data is organised as a two-level hierarchy to demonstrate the advantages of the CirGO software. Visualisation of GO terms using CirGO provides several major advantages. First, it is an intuitive prioritisation of the most significantly changing parent GO terms as the largest slices of the chart in order of significance. Comparable to tree-maps visualisation we present all input data, but in our CirGO implementation we can intuitively order and consequently single out the most significantly affected GO terms. The second major advantage is the use of parent and child parameters, which enables us to deposit multiple child GO terms related to a parent category. Thereby we remove overcrowding through listing every GO term as in a tree-map presentation. As a final advantage, the use of parent and child categories in our CirGO environment eliminates the overlap between common terms and the redundancy found on tree-map presentations.

To make tree-map plots and our visualisation approach comparable we prepared the tree-map input data (which is the output from REVIGO) in the same format as for the CirGO environment. The chosen number of visualised child categories (m) was limited to 40. In addition to the 40 desired child categories, 8 additional slices were included and labelled as “additional categories” to retain the same structure of the input data. As a result of using a space-filling technique we were successful in making use of the whole data and fit it into a defined 2D space. The tree-map in Fig. 3 was generated with Treemap R package [28], which was created with the in-house script available at [25]. Although, the provided space was utilised, the order of significance and priority of each category is not intuitively clear as in our CirGO visualisation (Fig. 1). Below we discuss additional improvements, which are provided by our CirGO visualisation software in visualising GO terms.

### Colour

Tree-maps apply various colours, where each colour indicates a parent term. Child terms have the same colour as the parent term. Our CirGO visualisation uses effective colour palettes generated by Colorgorical web-based tool that enables to distinguish parent terms from each other [29]. Moreover, child terms in CirGO employ gradient colours that are distributed from the darkest to lightest of the related parent colour to indicate the significance of the term from most to least significant, respectively. The gradient was created with Seaborn Python library [30]. Our CirGO visualisation also labels the parent slices with supporting labels “GO-number” that correlates the parent labels to the legend, and also as an indicator for colour vision impaired individuals.

### Values and shapes

The size of the rectangular shapes on tree-maps can be misinterpreted by users. The layout of the rectangles depends on the algorithm used to display the data. However, there is the same concern in the visualisation community regarding the size of the pie chart slices. To overcome this problem, our CirGO software is supporting numerical information located at the legend that helps to evaluate the size of each slice on the pie chart. In addition, child values are sorted from the most significant to the least. This helps to intuitively understand that the subsequent slice size is smaller than the previous one. Moreover, CirGO visualisation has an intuitive distribution of labels in a clockwise way, which is not supported by tree-maps. The rectangular flow of tree-maps is not as intuitive and depends highly on the applied algorithm.

### Font size

Tree-maps try to fit labels into the provided rectangular space that results in various labels and font size in one plot. There is an option that enables to retain the same font size, however this leads to the omission of some labels that do not fit into the provided space (Fig. 3). In contrast, CirGO displays all labels with the same font size providing consistency in publishing.

### Pros and cons

Tree-maps fill out the provided space entirely and present all the terms, albeit in inconsistent font and order of priority which can frequently use common and redundant categories. Although CirGO is a circular visualisation, the advantage of this visualisation lays in its intuitive ordering of prioritised parent categories and use of non-redundant terms.

## Conclusions

In summary, our CirGO visualisation has a two-layer hierarchy, where the inner circle represents a parent record or node. The outer circle is composed of one or more of the descendants of the parent, which are referred to as child records or nodes. Each category of the inner circle is reflected in the legend and supported with a related colour and text identifier “GO-number” catering to colour impaired individuals. Child nodes are organised as follows: each category of the child relates to one parent, that represents semantically similar term. These are sorted based on absolute log10 *p*-value from the smallest to largest, and a colour gradient is applied to highlight the largest to smallest value distribution. The GO analyses produce many final categories, which leads to cognitive overload if all of them are visualised. Therefore, we defined only a selected number of categories that are visualised. This number has to be pre-selected based on the underlying analysis data and can include up to 60 categories. To merge remaining categories under a certain value into one named “additional category”, we utilised thresholding. To demonstrate the strengths and advantage of our method, we have used a dataset of GO terms identified in a model of heart disease [31]. We visualised the most relevant and affected biological processes upon knockout of a gene, which is essential for survival and development (Fig. 1).

In conclusion, our Python-based open access CirGO software supports the visual analysis process through the representation of the most relevant GO terms in an appealing and intuitive way. A special feature of the tool is that it does not omit any related information. It creates an appealing visualisation, which easily fits within journal articles and other presentation formats. Succinctly it identifies the major findings from gene expression analyses. It is also very easy to use by scientists without any programming skills. Our CirGO software is a freely available Python package, eliminating the requirement for programming skills to visualise two-level hierarchical and redundant GO data in 2D format. It can be downloaded from [25]. Our CirGO software is licensed under the terms of the GNU general public license (version 3). This means it is a free software tool, which can be freely re-distributed and/or modified under the terms of the GNU General Public License v.3 as defined by the Free Software Foundation.

## Availability and requirements

**Project name:** CirGO

**Project home page:** https://github.com/IrinaVKuznetsova/CirGO.git

**Operating system(s):** Windows, Unix, MacOS

**Programming language:** Python 2.7+

**Other requirements:** NumPy 1.13.1, Matplotlib 2.1.0, Seaborn 0.8.1

**License:** GNU General Public License (version 3)

**Any restrictions to use by non-academics:** none

## Additional file

Additional file 1: The file contains the manual, directions and tuturial for the installation and use of the software described in this manuscript. (DOCX 515 kb)

## Abbreviations

GO: Gene ontology
RNA-Seq: RNA sequencing
