# Supplementary material for: CirGO: an alternative circular way of visualising gene ontology terms
Source: BMC Bioinformatics. 2019 Feb 18;20:84. doi: 10.1186/s12859-019-2671-2 (PMC6380029; doi:10.1186/s12859-019-2671-2)
Supplement: Supplementary file 1 — The file contains the manual, directions and tuturial for the installation and use of the software described in this manuscript. (DOCX 515 kb) [file 12859_2019_2671_MOESM1_ESM.docx]

Additional File for

CirGO: An alternative Circular way of visualising Gene Ontology terms

Irina Kuznetsova, Artur Lugmayr, Stefan Siira, Oliver Rackham and Aleksandra Filipovska

**Contents**

[1 The CirGO Algorithm 3](#_Toc536781940)

[1.1 Formatting Step (I) 5](#_Toc536781941)

[1.2 Values Calculation Step (II) 5](#_Toc536781942)

[1.3 Visualisation Step (III) 6](#_Toc536781943)

[2 Download Instructions and Required Software Packages 7](#_Toc536781944)

[2.1 Download Instructions 7](#_Toc536781945)

[2.2 Operating System (OS) 8](#_Toc536781946)

[2.3 Python Environment and Dependencies 8](#_Toc536781947)

[2.4 Operating Environments Where the Software Has Been Tested 10](#_Toc536781948)

[3 Installing and Running The CirGO Software Tool 10](#_Toc536781949)

[3.1 Step-By-Step Tutorial for Windows OS 10](#_Toc536781950)

[3.1.1 Installation Guide 10](#_Toc536781951)

[3.1.2 Package Usage 11](#_Toc536781952)

[3.2 Step-By-Step Tutorial for Unix/Linux 12](#_Toc536781953)

[3.2.1 Installation Guide 12](#_Toc536781954)

[3.2.2 Software Usage 13](#_Toc536781955)

[3.3 Step-By-Step Tutorial for Mac OS 13](#_Toc536781956)

[3.3.1 Installation Guide 13](#_Toc536781957)

[3.3.2 Software Usage 14](#_Toc536781958)

# The CirGO Algorithm

The CirGO visualisation algorithm consists of the following three basic workflow steps, which are also illustrated in Fig. 1:

I. Formatting step: converting and preparing a REVIGO file to a processing file;

II. Values calculation step: calculating, and organising values required for plotting;

III. Visualisation step: visualising the data and generating the final image.


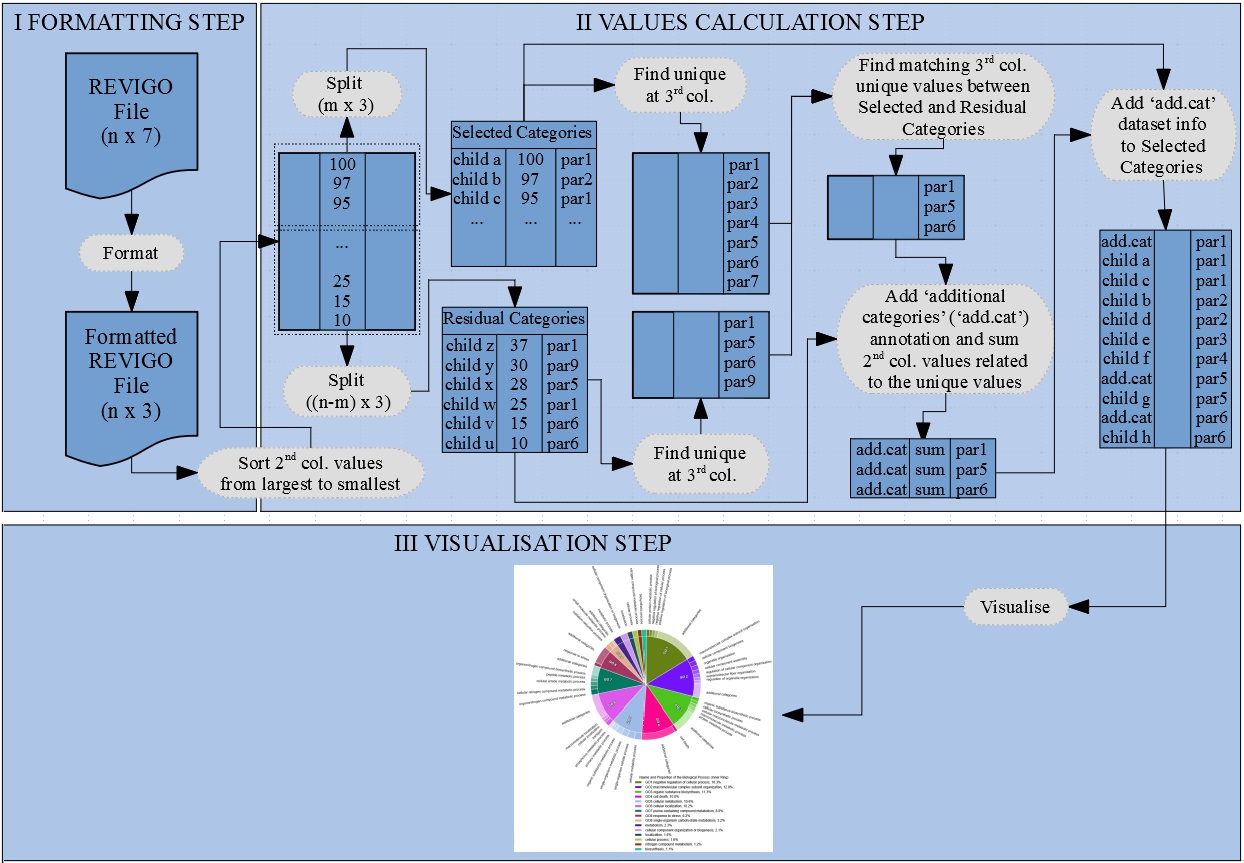


Fig. 1 Schematic representation of the steps in the CirGO workflow. The algorithm consists of three main steps: I. Formatting, II. Values calculation, and III. Visualisation. Main parameters are represented on the image as follows: n- represents number of input file rows; m- represents number of categories that will be visualised.

The algorithm converts a regular REVIGO file into an intermediate file, which is used to create an shown in Fig. 2. The final visualisation is a two-layer full hierarchical structure, and consists of an inner ring, and outer ring. The inner ring represents parent records, and the outer ring child records.


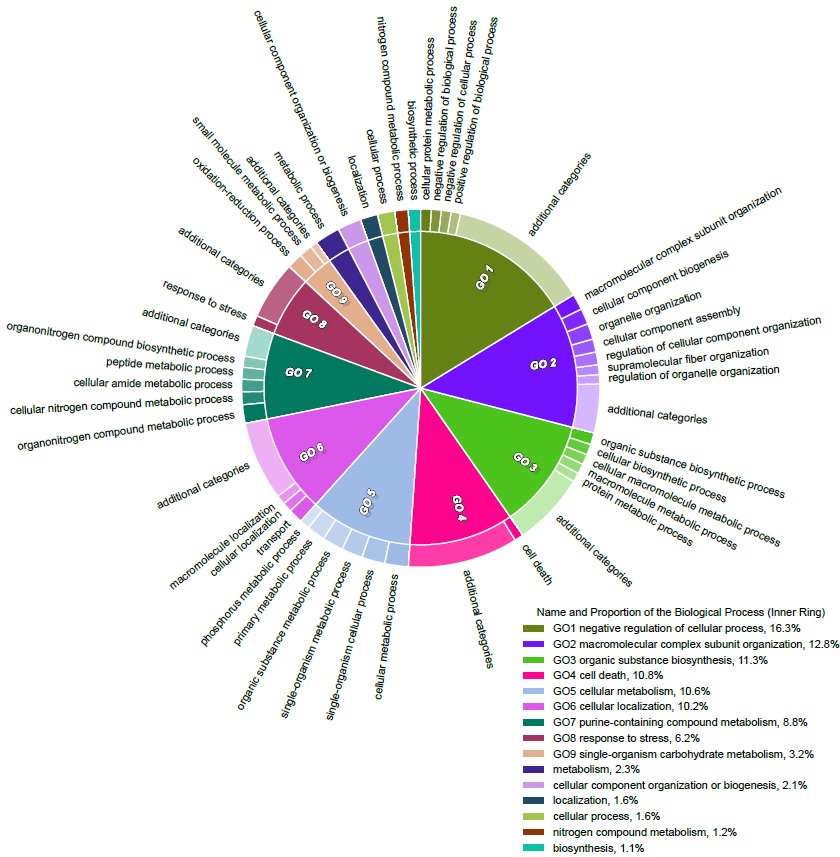


Fig. 2 Example of the CirGO image plot.

## Formatting Step **(**I)

During the Formatting Step (Fig. 1), the comma-separated values *(csv)* REVIGO output file (Table 1) is converted into a three columns tab delimited format (Table 2). A REVIGO *csv* file typically consists of seven columns. The initial four rows are commented with a “%” sign and followed by a header row. The converted file consists of a two-layer hierarchically structured data. The second, fourth, and seventh columns of the original REVIGO file named “description”, “log10pvalue”, and “representative”, are used to generate the image. The “description” column contains child records and is used to represent the outer ring. The “log10pvalue” converted to absolute values is used to represent the size of each parent record. The “representative” column contains parent records and is used as the inner ring component in the final visualisation. Please note, that columns with the text information may contain commas, which must be preserved and kept while parsing the *csv* input file.

*Table 1 Example of the REVIGO output file.*

*Table 2 Example of the formatted tab delimited three columns file.*

## Values Calculation Step (II)

This Value Calculation Step computes all required parameters, which are required for the plotting process. The main steps of this part of the workflow are:

(1) Parse the formatted three columns of the file produced by REVIGO software. For simplicity, the columns are designated as follows:

- the first column called “description” represents child records;
- the second column called “log10pvalue” defines slice sizes of the final circle in the visualisation;
- the third column called “representative” represents parent records.

(2) Select m, which magnifies the desired number of categories (or child terms) to be visualised.

(3) Sort data based on the “slice size” column from the largest to smallest. Subset the REVIGO output file data to m, the number of selected categories. Generate two new datasets:

- selected categories, representing the most significant number m of terms that should be visualised;
- residual categories, representing the data that is not included in the number of categories to be visualised (thus outside of the threshold m).

(4) Obtain unique categories from the “parent records” column for the “selected categories” subset.

(5) Obtain unique categories of the “parent records” column for the “residual categories” subset.

(6) Match a list of unique parent records (or inner ring) from the "selected categories" subset to a list of unique parent records from the “residual categories” subset. Sum-up all “slice size” values from the “residual categories” match these to the same “parent record” of the “selected categories”, and assign “child records” as “additional categories”.

(7) Combine information obtained at step (6) with the “selected categories” subset. This information includes labels for the inner and outer rings, and slice sizes for both rings.

## Visualisation Step (III)

The Visualisation Step combines all required information for generating an image in a scalable vector graphics (*svg*) format. The main steps of the visualisation step are:

(1) Generate a list of hex encoded colours. To obtain effective colour palettes use Colorgorical web-based tool (vrl.cs.brown.edu/color).

(2) Calculate and assign colour values to the inner and outer rings, where the outer ring colours are represented as a gradient of the inner ring colour cluster. The outer ring colours are represented from the darkest to brightest, highlighting the distribution of the most significant to the least significant categories.

(3) Calculate supporting numerical information of wedges size (a slice proportion). This information is represented as percentage in the legend.

(4) Initialise parameters required for plotting the outer ring.

(5) Obtain the text identifier “GO-number” that is placed in the inner ring. This number is used to support colour impaired individuals with the information between the legend and the image. Please note, if a size of the wedge is less than 3%, the “GO-number” identifier is not visualised.

(6) Initialise parameters required for plotting the inner ring.

(7) Generate a legend that shows the inner ring labels information and identifies a wedge size as a percentage.

(8) Save the image in *svg* format.

# Download Instructions and Required Software Packages

## **Download Instructions**

The CirGO software is freely available and can be downloaded from the GitHub page <https://github.com/IrinaVKuznetsova/CirGO.git>.

## Operating System (OS)

The CirGO software will run on Windows, Unix\Linux, or Mac OS in three different modes, which can be selected as command line parameter:

I. Interactive Graphical User Interface (GUI) mode;

II. Command-Line Interface (CMD) mode;

III. Interactive Command-Line Interface (INT) mode.

## Python Environment and Dependencies

CirGO was implemented as a Python package and is using the Python 2.7.14, Matplotlib 2.1.0, and the GUI components using the TkInter (Tcl/Tk) package. Table 3 shows packages required to run the software, including their different versions:

*Table 3. Python environment and dependencies.*

| **Software Package Name, and Version** | **Description** | **Link** |
| --- | --- | --- |
| **Windows** | | |
| Python 2.7.13 | Programming language. | <https://www.python.org/download/releases/2.7/> |
| Anaconda Distribution | Python distribution environment that contains package management tools. | <https://www.anaconda.com/download/> |
| NumPy 1.13.1 | Array-processing package used for scientific computing with Python for numbers, strings, records, and objects. | <https://pypi.python.org/pypi/numpy/1.13.1> |
| Matplotlib 2.1.0 | Python library used for generating 2D images. | <https://matplotlib.org/2.1.0/api/index.html> |
| Seaborn 0.8.1 | Python visualisation library. | https://seaborn.pydata.org/ |
| TkInter  $Revision: 81008 | Python package used for writing a Graphical User Interface (GUI). | <https://wiki.python.org/moin/TkInter> |
| Argparse 1.1 | Python module used for producing a user-friendly command-line interface. | <https://docs.python.org/3/library/argparse.html> |
| **Unix/Linux** | | |
| Python 2.7.6 | Programming language. | <https://www.python.org/download/releases/2.7/> |
| Virtualenv 15.1.0 | Tool for creating Python isolated virtual environment. | <https://virtualenv.pypa.io/en/stable/> |
| NumPy 1.13.1 | Array-processing package used for scientific computing with Python for numbers, strings, records, and objects. | <https://pypi.python.org/pypi/numpy/1.13.1> |
| Matplotlib 2.1.0 | Python library used for generating 2D images. | <https://matplotlib.org/2.1.0/api/index.html> |
| Seaborn 0.8.1 | Python visualisation library. | https://seaborn.pydata.org/ |
| TkInter  $Revision: 81008 | Python package used for writing a Graphical User Interface (GUI). | <https://wiki.python.org/moin/TkInter> |
| Argparse 1.1 | Python module used for producing a user-friendly command-line interface. | <https://docs.python.org/3/library/argparse.html> |
| **Mac OS** | | |
| Python 2.7.10 | Programming language. | <https://www.python.org/download/releases/2.7/> |
| Virtualenv 15.1.0 | Tool for creating Python isolated virtual environment. | <https://virtualenv.pypa.io/en/stable/> |
| NumPy 1.13.1 | Array-processing package used for scientific computing with Python for numbers, strings, records, and objects. | <https://pypi.python.org/pypi/numpy/1.13.1> |
| Matplotlib 2.1.0 | Python library used for generating 2D images. | <https://matplotlib.org/2.1.0/api/index.html> |
| Seaborn 0.8.1 | Python visualisation library. | https://seaborn.pydata.org/ |
| TkInter  $Revision: 81008 | Python package used for writing a Graphical User Interface (GUI). | <https://wiki.python.org/moin/TkInter> |
| Argparse 1.1 | Python module used for producing a user-friendly command-line interface. | <https://docs.python.org/3/library/argparse.html> |

## Operating Environments Where the Software Has Been Tested

Table 4 shows the OS where the CirGO software has been tested.

*Table 4. Operating environments, where the software has been tested.*

| **OS** | **Version** |
| --- | --- |
| Windows | Windows 7 Professional |
|  | Windows 10 |
| Unix/Linux | 3.19.0-49-generic #55~14.04.1-Ubuntu SMP Fri Jan 22 11:24:31 UTC 2016 x86_64 x86_64 x86_64 GNU/Linux |
| Mac OS | OS X Yosemite Version 10.10.5 |
|  | OS High Sierra Version 10.18.3 |

# Installing and Running The CirGO Software Tool

## Step-By-Step Tutorial for Windows OS

### Installation Guide

An Anaconda distribution, also called Anaconda Prompt, is required to be installed on Windows OS under Python 2.7 (<https://www.anaconda.com/download/>).

1. Download [Anaconda distribution](https://www.anaconda.com/download/) for Python 2.7 on your Windows machine.
2. Open the Anaconda Prompt terminal.
3. Create a virtual environment. Virtual environments act as an isolated platform where you can install all required packages for the specific project, without modifying existing projects. For example, if the computer has Python 2.7+, Python 3+ can be installed without affecting Python 2+ environments.

> conda create --name SelectEnvirName

1. Activate created virtual environment. Check installed packages. Please note, an empty environment has no installed packages. A *pip* tool, which is a package manager, should be installed, enabling installation of required software packages. It is important to verify packages that have been installed.

> activate SelectEnvirName

(SelectEnvirName)> conda list # new just created environment has no installed packages

(SelectEnvirName)> conda install pip

(SelectEnvirName)> conda list

Please note, if *conda install pip* command offers Python3.7+, reply “*n*” to the subsequent question, deactivate and delete the created environment and run *conda create -n SelectEnvirName python=2.7* command. More details about this process can be found at the *conda* documentation page (https://conda.io/docs/user-guide/tasks/manage-environments.html#removing-an-environment).

(SelectEnvirName)> conda deactivate # Deactivate created environment

> conda env remove --name SelectEnvirName # Delete created environment

> conda create -n SelectEnvirName python=2.7 # Install a new environment that contains Python2.7+

> activate SelectEnvirName # Activate created environment

(SelectEnvirName)> conda list # Check installed Python version

1. Download the CirGO software from the GitHub repository as a zip folder (*Clone or Download*; *Download ZIP*). Unzip this folder into a directory of your choice, for example, to the desktop.
2. Navigate to the *setup.py* script of the CirGO software, which is located in the *docs* folder.

(SelectEnvirName)> cd YOURPATH\CirGO-master\docs

1. Create a source distribution for the CirGO package. This step generates two folders *CirGO.egg-info* and *dist*.

(SelectEnvirName)> python setup.py sdist

1. Navigate to the *dist* folder and install the CirGO package.

(SelectEnvirName)> cd dist

(SelectEnvirName)> pip install CirGO-0.1.0.tar.gz

### Package Usage

1. Navigate to the **CirGO_Wind_Unix** folder.

(SelectEnvirName)> cd YourPath\...\CirGO-master\CirGO_Wind_Unix

1. CirGO can be executed as a Graphical User Interface (GUI), Command-Line (CMD) or Interactive Command-Line (INT) mode on the Windows OS. Please note, provide a file directory as a string, where PATH backslashes '\' have to be changed to forward slashes '/' in Windows environment. To use the package type one of the commands in the terminal:

(SelectEnvirName)> Python CirGO.py -h # help page

(SelectEnvirName)> Python CirGO.py -gui # graphical user interface

(SelectEnvirName)> Python CirGO.py -h # command line

(SelectEnvirName)> Python CirGO.py -int # interactive command-line interfaces

## Step-By-Step Tutorial for Unix/Linux

### Installation Guide

1. Open the Unix/Linux terminal.
2. Install the *virtualenv* tool on your Unix/Linux terminal if you have not installed it already. This tool allows to create an isolated Python environment.

$ pip install virtualenv

1. Install the *pip* tool if you have not installed it yet. Pip is designed for managing software written in Python.

$ sudo apt-get install python-pip

1. Create a virtual environment. Virtual environments act as an isolated platform where you can install all required packages for the specific project without modifying existing projects. For example, if your computer has Python 2.7, you can easily install Python 3+ without affecting Python 2+ environments.

$ virtualenv SelectEnvirName

1. Activate created virtual environment.

$ source SelectEnvirName/bin/activate

1. Download the CirGO software from the GitHub repository as a zip folder (*Clone or Download*; *Download ZIP*). Unzip this folder into a directory of your choice, for example, to the desktop.
2. Navigate to the *setup.py* script of the CirGO software, which is located in the *docs* folder.

(SelectEnvirName)$ cd YOURPATH\CirGO-master\docs

1. Create a source distribution for the CirGO package. This step generates two folders *CirGO.egg-info* and *dist*.

(SelectEnvirName)$ python setup.py sdist

1. Navigate to the *dist* folder and install the CirGO package.

(SelectEnvirName)$ cd dist

(SelectEnvirName)$ pip install CirGO-0.1.0.tar.gz

### Software Usage

1. Navigate to the **CirGO_Wind_Unix** folder.

(SelectEnvirName)$ cd YourPath\...\CirGO-master\CirGO_Wind_Unix

1. CirGO can be executed as a Graphical User Interface (GUI), Command-Line (CMD) or Interactive Command-Line (INT) mode on Unix. To use the desired interface, type one of the commands in the terminal:

(SelectEnvirName)$ python CirGO.py -h # help page

(SelectEnvirName)$ python CirGO.py -gui # graphical user interface

(SelectEnvirName)$ python CirGO.py -cmd # command line

(SelectEnvirName)$ python CirGO.py -int # interactive command-line interfaces

## Step-By-Step Tutorial for Mac OS

### Installation Guide

1. Open the Mac terminal.
2. Install the *xcode-select* utility on your Mac OS if you have not previously installed it. These utilities enable use of common Unix-based tools.

$ xcode-select –-install

1. Install [*Homebrew*](https://brew.sh/) (https://brew.sh/), which is a free open source software package, if you have not previously installed it. It facilitates a software installation on Mac OS. Please note, always check the command from the [Homebrew](https://brew.sh/) website, as it is frequently modified.

$ /usr/bin/ruby -e "$(curl –fsSL https://raw.githubusercontent.com/Homebrew/install/master/install)"

1. Check Python version. Python 2+ version is required for the CirGO software. Please note, Python 2.7 generally comes with the Mac OS.

$ python --version

1. Create a virtual environment. Virtual environment acts as an isolated platform where you can install all required packages for the specific project without modifying existing projects. For example, if your computer has Python 2.7, you can easily install Python 3+ without affecting Python 2+ environments.

$ virtualenv -p /usr/bin/python2.7 SelectEnvirName

1. Activate the created virtual environment.

$ source SelectEnvirName/bin/activate

1. Install the latest *pip* version.

(SelectEnvirName)$ curl https://bootstrap.pypa.io/get-pip.py | python

1. Download the CirGO software from the GitHub repository as a zip folder (*Clone or Download*; *Download ZIP*). Unzip this folder into a directory of your choice, for example, to the desktop.
2. Navigate to the *setup.py* script of the CirGO software, which is located in the *docs* folder.

(SelectEnvirName)$ cd YOURPATH\CirGO-master\docs

1. Create a source distribution for the CirGO package. This step generates two folders *CirGO.egg-info* and *dist*.

(SelectEnvirName)$ python setup.py sdist

1. Navigate to the *dist* folder and install the CirGO package.

(SelectEnvirName)$ cd dist

(SelectEnvirName)$ pip install CirGO-0.1.0.tar.gz

### Software Usage

CirGO can be executed in three modes – as a Graphical User Interface (GUI), Command-Line (CMD) or Interactive Command-Line (INT) on Mac OS. To use the desired package type, use one of the command line operators in the terminal.

1. Navigate to the **CirGO_Mac** folder.

(SelectEnvirName)$ cd YourPath\...\CirGO-master\CirGO_Mac

1. CirGO can be executed as a Graphical User Interface (GUI), Command-Line (CMD) or Interactive Command-Line (INT) modes on Mac OS. To use the package type one of the commands in the terminal:

(SelectEnvirName)$ python CirGO.py -h # help page

(SelectEnvirName)$ python CirGO.py -gui # graphical user interface

(SelectEnvirName)$ python CirGO.py -cmd # command line

(SelectEnvirName)$ python CirGO.py -int # interactive command-line interfaces
